# Panx1 and drug discovery: from non-specific inhibitors to tailored drug compounds

**DOI:** 10.1007/s00441-025-04038-1

**Published:** 2026-01-16

**Authors:** Michael Kohr, Carola Meier

**Affiliations:** Department of Anatomy and Cell Biology, Medical Faculty, Saarland University, Kirrberger Strasse 100, Building 61, Homburg / Saar, Germany

**Keywords:** Pannexin 1, ATP, Inhibitor, Drug discovery, Channel, Selectivity

## Abstract

Pannexin (Panx)-1-mediated ATP release has been associated with a broad range of pathological conditions. Conversely, the use of Panx1-inhibitors has shown promising results in the medication of diseases, such as neuroinflammation, melanoma and epilepsy. In addition, Panx1-inhibitors are an indispensable tool for the elucidation of both structure and physiology of Panx1. Over the past years, numerous applications of Panx1-inhibitors have led to new insights into Panx1 influences in health and disease. The major drawback of conventional Panx1-inhibitors, however, is the lack of selectivity resulting in undesired side effects. Nevertheless, these inhibitors are useful resources for drug discovery and lead optimisation approaches have therefore found their way into Panx1 research. Newly developed inhibitors show both high efficacy and selectivity. The combination of drug development and molecular dynamics simulations is a powerful tool to further elucidate both structure and gating mechanisms of Panx1.

## Introduction

Adenosine triphosphate (ATP) is a ubiquitously found, highly energetic biochemical compound of the cytosol, that is required for energy-consuming processes in both physiological and pathological contexts. The mechanisms of ATP release from the cell cytoplasm into the extracellular space is therefore carefully regulated by gating proteins. The pannexin (Panx) transmembrane protein family has gained increasing attention as a major ATP release channel and to date three isoforms have been discovered: Panx1 is by far the most prominent isoform and is ubiquitously expressed, while Panx2 although initially believed to be restricted to the CNS (Bruzzone et al. 2003; Baranova et al. 2004; Zoidl et al. 2008) has since been detected in peripheral tissues e.g. in the blood vasculature, in smooth muscle and epidermis (Le Vasseur et al. 2014; Diezmos et al. 2015; Sanchez-Pupo et al. 2022; O’Donnell et al. 2025). Panx3 expression is more selective and can be found in the musculoskeletal system (O’Donnell and Penuela 2021). Notably, pannexins are already expressed at early stages of development, for instance in neuronal ganglia of murine embryos and in human pluripotent stem cells (Raslan et al. 2016; Hainz et al. 2018). Due to their sequence homology with the invertebrate gap junction protein family of innexins, pannexins were initially suspected to form gap junctions, which was disproven by independent methods e.g. morphology studies by electron microscopy (Boassa et al. 2007; Beckmann et al. 2016). Increased Panx1-mediated ATP release correlates with a range of pathological conditions including multiple sclerosis, melanoma and epilepsy (Santiago et al. 2011; Penuela et al. 2012; Lutz et al. 2013), but then again ATP release has a tumour-suppressive effect on glioma cells (Lai et al. 2007) and attenuates *Pseudomonas aeruginosa* infections when released from tuft cells (Abdel Wadood et al. 2025).

**Fig. 1:**
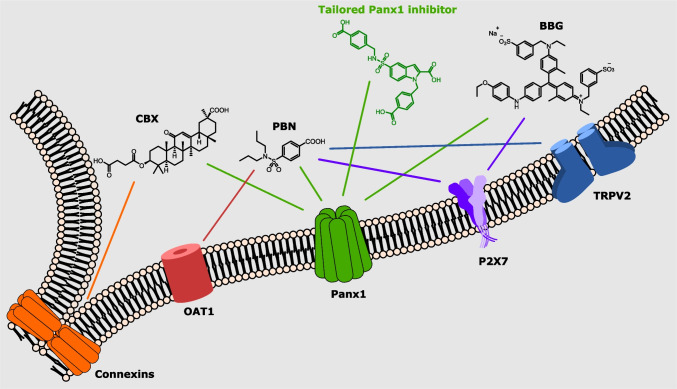
Panx1 (green) and four potential side targets Connexins (orange), OAT1 (red), P2X_7_ (purple) and TRPV2 (blue) of the displayed Panx1-inhibitors CBX, PBN and BBG are shown embedded in a phospholipid double layer. The coloured lines assign inhibitors to their respective (side) targets. The tailored Panx1 inhibitor is displayed in green colour

These examples demonstrate the complexity of ATP release and action, and sophisticated tools are necessary to illuminate the regulatory mechanisms of Panx1. Panx1-inhibitors have been valuable tools to modulate ATP release in cells and tissues, for instance reducing ATP release of macrophages in acute *Pseudomonas aeruginosa* pneumonia (Wonnenberg et al. 2014). In addition, Panx1-inhibitors have also contributed to elucidate Panx1 structure. In 2020, a carbenoxolone (CBX)-bound Panx1 cryo-electron microscopy (EM) structure revealed a putative binding site of Panx1-inhibitors, which may also play a significant role in physiological gating mechanisms (Ruan et al. 2020). A combination of electrophysiology studies, ATP release assays and dye uptake assays has helped to identify numerous inhibitors. However, most of these inhibitors are common drugs, not specifically developed to target Panx1 (Fig. 1). Thus, their major drawback is the lack of selectivity for Panx1 over other targets, which complicates the interpretation of biological results and generates hazardous side effects. In view of the increasing number of known physiological and pathological processes involving Panx1, the need for specific inhibitors is growing steadily. Today, more than 30 repurposed Panx1-inhibitors have been discovered with most of them containing individual structural motifs. This structural diversity gives plenty of options to develop optimised inhibitors and, unfortunately, the complexity of such an endeavour may seem invincible without any point of reference. In this perspective, the discovery of the Panx1 structure by cryo-EM in 2020 has been a breakthrough, leading to the development of the first tailored Panx1-inhibitors. This review summarises the most recent development of Panx1-inhibitor research including applications of well-established Panx1-inhibitors, new “recycled” inhibitors as well as drug discovery.

## “Recycled” Panx1-inhibitors

**Fig. 2:**
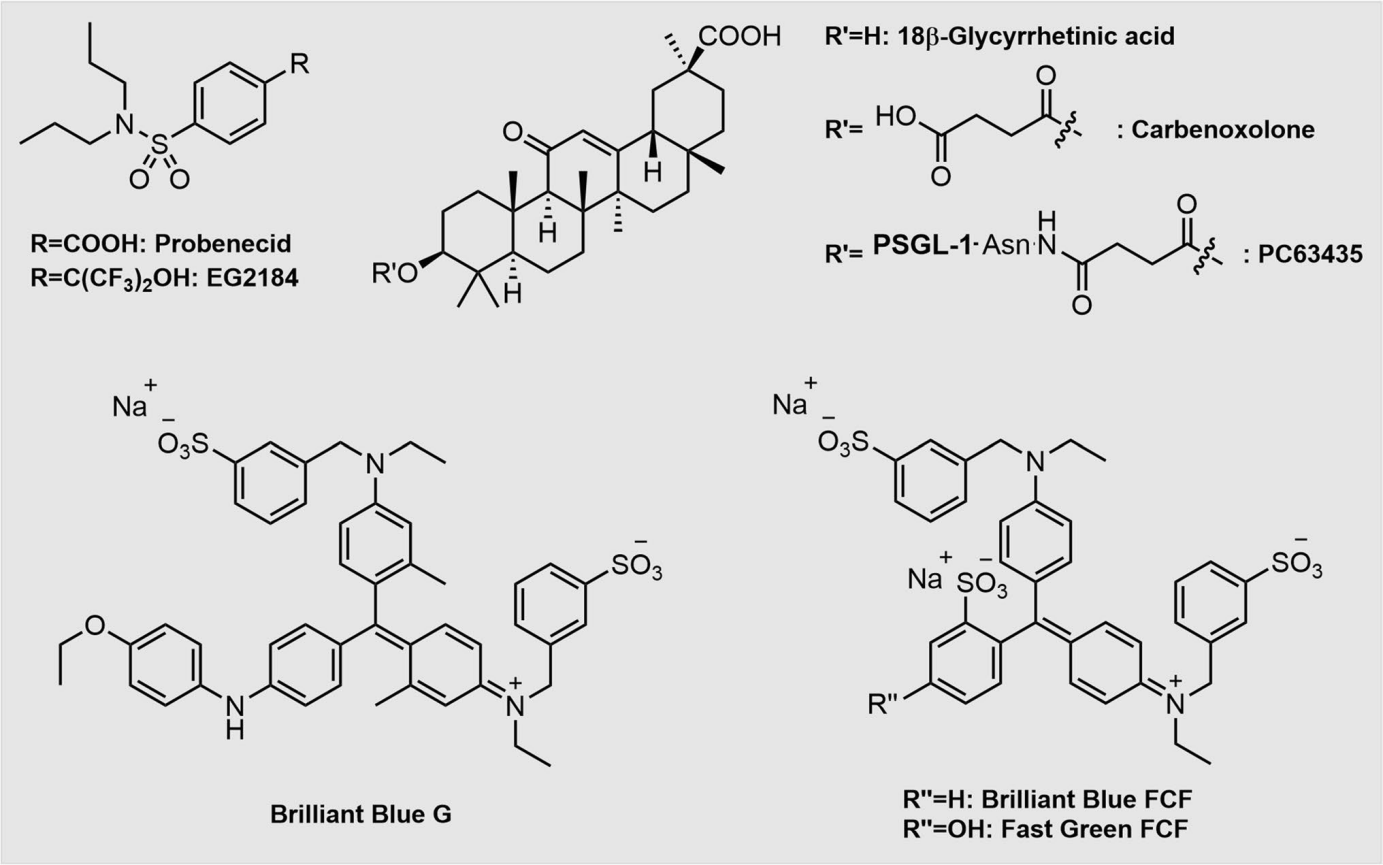
Chemical structures of the three most frequently applied “recycled” Panx1-inhibitors and their derivatives. PBN and the brilliant blue dyes contain aromatic residues allowing them to interact with Panx1 by π-stacking. CBX on the other hand has a steroid-type core structure without aromatic systems. BB-FCF and Fast Green FCF share common structural motifs and only differ by one hydroxy group

Over the past two decades, various substances were reported to inhibit Panx1 channels, however, most of them had initially been characterised as inhibitors of other targets. It is therefore not surprising that most of these agents are not solely selective to Panx1. Yet, these inhibitors, and particularly the combination of two or more orthogonal inhibitors, have been utilised to gain further insights into physiological functions, structure and regulation of Panx1. While some have gained high popularity in Panx1 research, others have not been given much attention, partly because they lack selectivity, efficacy or they are simply expensive. Nevertheless, besides the well-established Panx1-inhibitors (Fig. 2), lesser-known compounds also deserve attention and some of them will be mentioned briefly. Even if a particular Panx1-inhibitor does not bring the ideal properties, which justify its application in Panx1 research, it may still act as a starting point for further lead optimisations in Medicinal Chemistry research. Surprisingly, drug discovery had not found its way into pannexin research for a long time but within the last five years, tremendous efforts have been taken to develop new Panx1-specific inhibitors, most of which are in fact based on traditional Panx1-inhibitors.

### Probenecid

Probenecid (PBN), a pharmacologically active compound and approved drug, is widely known as a gout remedy (Fig. 2). Yet, since the discovery of its ability to elevate serum penicillin concentrations (Burnell and Kirby 1951), it has been in the centre of a broad range of applications. PBN even gained attention in the field of sports as it entered the WADA’s (World Anti-Doping Agency) list of prohibited substances and methods, where it is considered a masking agent. Nowadays, PBN has widely been replaced since more effective gout therapies had been discovered (Rider and Jordan 2010). Over the last 20 years, PBN has regained attention for being an inhibitor as well as an activator of certain transmembrane proteins. The inhibitory effect of PBN on Panx1 channels was revealed for the first time by patch-clamp experiments with *Xenopus laevis* oocytes (Silverman et al. 2008) and the results were confirmed by dye-uptake and ATP release assays. PBN’s main benefit compared to other Panx1-inhibitors, is the ability to differentiate entirely between pannexins and connexins, which has made it one of the most popular reagents for the research of pannexin channels. Despite its selectivity to Panx1 over connexin channels, PBN does in fact interact significantly with other targets. OAT1 and OAT3 are two members of the organic anion transporter (OAT) family and their interaction with PBN is the main reason for its uricosuric effects (Selen et al. 1982). Besides OAT, the transient receptor potential vanilloid (TRPV) 2 is another important target of PBN. The ion channel was demonstrated to be blocked by PBN in cultured mouse trigeminal neurons (Bang et al. 2007). Even organic cation transporters (OCT) are among PBN’s suspected targets, as PBN affected the uptake of cimetidine (McKinney et al. 1981) and renal excretion of famotidine (Inotsume et al. 1990). PBN also affects the purinergic P2X_7_ receptor, however, inhibition seems to occur via a Panx1-independent mechanism (Bhaskaracharya et al. 2014). Regarding the application of PBN in neurological disorders, it is of importance that PBN can pass the blood–brain-barrier (Qi et al. 2015). As such, PBN attenuates symptoms caused either by experimental autoimmune encephalomyelitis (EAE) and by cuprizone-induced demyelination in mice (Hainz et al. 2017a, b).

In the recent past, PBN has remained a prominent agent for pannexin channel research and the effect of Panx1 on various diseases has been under examination in vitro and in vivo, including in colon cancer progression (Fierro-Arenas et al. 2024), in ileal ischemia and reperfusion (I/R) (Pereira et al. 2025), autosomal dominant polycystic kidney disease (ADPKD) (Arkhipov et al. 2023), in neuroblastoma treatment (Langlois et al. 2023) and in sepsis (Meng et al. 2025). Another interesting example is the use of PBN to treat alcoholic disease (Hornbacher et al. 2024). PBN was able to reduce alcohol urge and craving in individuals, who regularly consume alcohol, when PBN was co-administered with alcohol. The authors presumed that alcohol-mediated increase of extracellular adenosine may be related to alcohol craving and is therefore counteracted by PBN-mediated Panx1-inhibition. In the context of opioid withdrawal studies (Kwok et al. 2024), PBN was used to attenuate the physical signs of naloxone-precipitated withdrawal as well as opioid withdrawal-induced conditioned place aversion (CPA), however, high doses of PBN were necessary to trigger the desired effects. Consequently, the group synthesised the compound EG-2184, a PBN derivative with a hexafluoryl alcohol in place of the carboxylic acid group (Fig. 2). The new functional group was responsible for a higher lipophilicity and inhibited Panx1-mediated Yo-Pro-1 uptake at a 0.01 µM concentration. In HEK-293 T cells, EG-2184 was more potent than PBN (EG-2184: IC_50_ = 54 nM, PBN: IC_50_ = 74.6 µM) with respect to Panx1-mediated current inhibition. In vivo, the symptoms of drug withdrawal were attenuated with similar tendencies for both PBN and EG-2184. Importantly, EG-2184 did not block P2X_7_R-mediated calcium responses at 10 nM, while PBN was not able to differentiate significantly between Panx1 and P2X_7_R.

### Carbenoxolone

Carbenoxolone (CBX), a glycyrrhetinic acid derivative, was originally developed to overcome the limited water solubility of most other derivatives, which were known to have promising anti-inflammatory effects. In 1959, the anti-inflammatory effects and the solubility of CBX were investigated for the first time (Finney and Tárnoky 1960). Since then, CBX has gained in popularity for the treatment of inflammatory gastrointestinal diseases as well as its activity against gap junction proteins. CBX’s activity on Panx1 was discovered shortly after the discovery of pannexins (Bruzzone et al. 2005). Today, CBX is suspected to bind Panx1 in the first extracellular loop, where a ring of seven W74 from different protomers can be found (Ruan et al. 2020). Interestingly, CBX is special in that it is structurally not able to interact by π-stacking, a non-covalent interaction between two aromatic systems creating a double-layered structure.

CBX has been crucial to further reveal the structure of Panx1 and it is nowadays still used alongside other compounds as a Panx1-inhibitor in physiological studies, for instance to elucidate the synergy between the NMDA receptor and Panx1 (Zepeda-Morales et al. 2025). The group underscored the activation of Panx1 by NMDAR-induced Src kinase in nerve-injured rats. C-terminal truncation of Panx1 is generally suspected to cause cell death and the correlation with calcium influx has recently been under investigation (Salgado et al. 2024). In this context, both ^10^Panx1 (see chapter “Medicinal Chemistry in Panx1 research” for a detailed discussion about ^10^Panx1) and CBX prevented cell death by inhibiting Panx1. Similarly, both inhibitors were utilised to examine the effect of Panx1-inhibition on central post-stroke pain (CPSP) (Bu et al. 2023), concluding that Panx1 inhibition was the decisive factor. However, the combination of CBX and ^10^Panx1 should always be considered carefully as they are known for interferences with connexins. Thus, a third orthogonal inhibitor would have been desirable to exclude connexins as a side target.

The involvement of Panx1 in the NLRP3 inflammasome is a complicated topic and cannot be fully covered in this review (Huang et al. 2024; Soylu et al. 2025; Bravo et al. 2025). Yet, it should be briefly mentioned that CBX has been utilised to investigate influences of Panx1 and ATP release in ischemia/reperfusion (I/R)-induced acute kidney injury (AKI) (Yin et al. 2022). An innovative approach to target Panx1 in the context of platelet formation is the combination of CBX and CD62P ligands. Moreover, Li et al. combined the affinity of CBX to Panx1 with P-selectin glycoprotein ligand-1 (PSGL-1) and interconnected both by chemical ligation (Fig. 2). The resulting Panx1-inhibitor PC63435 was thereafter injected into WT mice, which caused prolonged bleeding times and impaired platelet aggregation without altering the platelet counts (Li et al. 2023). CBX has proven itself to be a versatile tool in Panx1 research, albeit with some undesirable features. Its major drawback is the relatively low selectivity towards pannexin channels. Although the compound can discriminate between pannexins and connexins to some extent, this differentiation process is highly dependent on the applied dose (Silverman et al. 2008). Nevertheless, the combination of CBX together with PBN is a great method to rule out P2X_7_R and connexin related effects in Panx1 research.

### Brilliant blue dyes

Brilliant Blue G (BBG) was discovered to act as a Panx1-inhibitor alongside ATP and BzATP (Qiu and Dahl 2009). Intriguingly, the use of an R75 mutant of Panx1 attenuated BBG inhibition, indicating that BBG may share similar modes of action with PBN and CBX. Whereas BBG is also an inhibitor of the human P2X_7_ receptor (but not of connexins), the omnipresent food dye BB-FCF, a BBG analogue with a slightly different substitution pattern, is more Panx1-specific and a substantially stronger inhibitor (BB-FCF: IC_50_ = 0.27 µM vs. BBG: IC_50_ = 3 µM) (Wang et al. 2013). Identical efficacies could also be observed for Fast Green FCF, which structurally only differs from BB-FCF by one hydroxy group (Fig. 2). Both mutation experiments (Wang et al. 2013) and cryo-EM data (Ruan et al. 2020) suggest W74 as part of the selectivity filter.

Recently, BBG and BB-FCF have been used for inhibition of Panx1 or P2X receptors in biological studies. Panx1-mediated ATP-release into the lumen of the urinary bladder i.e. from the apical surface of the urothelium, but not from the basolateral surface (BB-FCF) could be evaluated (Beckel et al. 2015). BBG further helped to investigate the effects of ischemia followed by reperfusion onto enteric glial cells (EGCs). In this context, BBG and PBN were able to induce the recovery of GFAP-positive EGCs and neurons (Mendes et al. 2023). Additionally, BBG was applied as a therapeutic agent for the treatment of muscle diseases caused by SARS-CoV-2 in a mouse model (Gouvêa De Souza et al. 2025). Panx1-inhibitors including BB-FCF were also utilised to investigate the correlation of fluid shear stress and ATP release (Verschuren et al. 2020), for instance resulting in reduced renal cyst growth in mice.

### Trovafloxacin

**Fig. 3:**
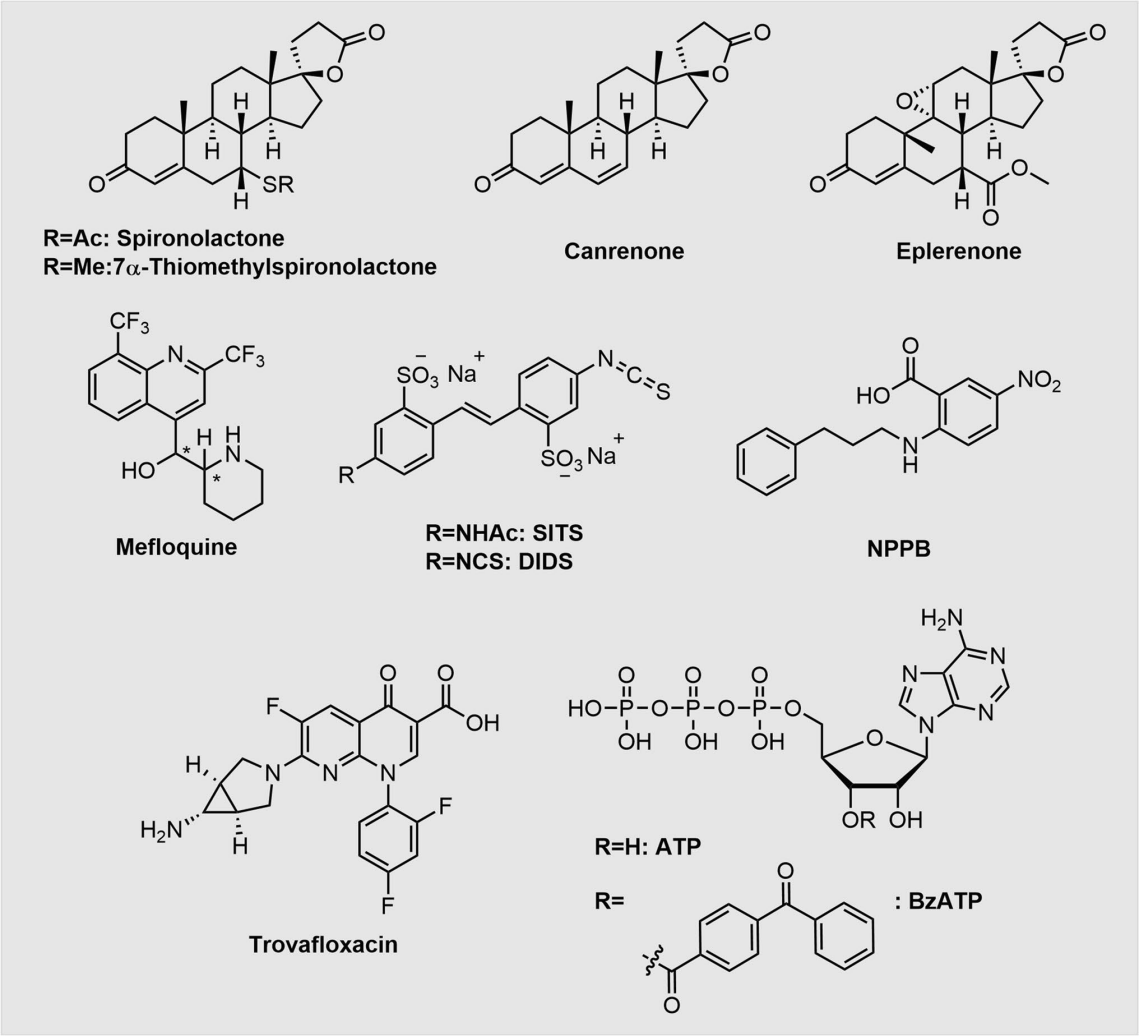
Chemical structure of some established Panx1-inhibitors and derivatives thereof. Most inhibitors contain aromatic systems or a steroid-type structures. Acidic groups are suspected to interact with R75 in the extracellular loop and carboxylic acid moieties can be found in trovafloxacin and NPPB, whereas sulfonic or phosphoric acids are abundant in SITS, DIDS, ATP and BzATP. The spironolactone derivatives and mefloquine do not have a typical acidic group and different binding modes may seem plausible

The naphthyridone CP-99,219, also known as trovafloxacin or Trovan (Fig. 3), was discovered more than thirty years ago (Gooding and Jones 1993) and is a more potent antibiotic than structurally related compounds such as ciprofloxacin or ofloxacin. In 1999, however, it was reported that during post market surveillance of trovafloxacin 140 out of 2.5 million patients, being treated with trovafloxacin, displayed serious hepatotoxic symptoms including cases of required liver transplantation (Ball et al. 1999). In consequence, the drug was taken off the market shortly after. Trovafloxacin eventually regained attention in 2014 after a screening of 1280 small molecules on Panx1-inhibitory activity confirmed its influence on Panx1 (Poon et al. 2014). Although trovafloxacin exhibited significantly higher toxicities than levofloxacin (Kaden et al. 2023), it remains an interesting drug compound due to its superior selectivity towards Panx1 while targeting neither Panx2 nor Cx43 (Poon et al. 2014). In addition, trovafloxacin did not affect P2X_7_R as no influence on BzATP-induced dye uptake was observed (Adamczyk et al. 2015). In initial screenings, Trovan derivatives like ciprofloxacin and levofloxacin did not block Panx1-dependent dye uptake. In contrast, difloxacin and tosufloxacin partially inhibited ATP release and TO-PRO-3 uptake by apoptotic cells, albeit less potently than trovafloxacin. Unfortunately, trovafloxacin has only rarely been used in Panx1 research over the last few years, presumably due to its high price and complex structure compared to PBN or CBX. However, there are reports about the use of trovafloxacin, for instance to illuminate the role of Panx1 in the regulation of the size of apoptotic bodies (ApoBDs) (Phan et al. 2021). Another group (Giustarini et al. 2019) harnessed the hepatotoxic properties of trovafloxacin to gain further insights into mechanisms of DILI (drug-induced liver injury). Beneficial effects of trovafloxacin on the outcome of traumatic brain injury, particularly reduction of inflammation and brain damage, were described in a murine controlled cortical impact (CCI) model (Garg et al. 2018). The role of Panx1 in the promotion of effector CD8^+^ T cell responses (Vardam-Kaur et al. 2024) and the correlation of Panx1-inhibition with focal laser ablation (FLA)-induced flashing ATP release in the brain (Chen et al. 2024) had also been investigated by using trovafloxacin.

### Spironolactone and metabolites

In 2018, spironolactone was identified as a Panx1-inhibitor after an unbiased small molecule screening (Good et al. 2018), showing dose-dependent inhibition of Panx1 in HEK293 T cells (IC_50_ = 7–8 µM). The compound was originally developed as an antihypertensive drug and designed as an antagonist of the mineralocorticoid receptor NR3C2 (Karim 1978). Notably, spironolactone also affects α1-adrenergic arteriolar vasoconstriction and blood pressure in mice, which is independent of NR3C2. Canrenone and 7α-thiomethylspironolactone (Fig. 3), two metabolites of spironolactone, also inhibit Panx1 currents but not recombinant Panx2 (Good et al. 2018). Similar effects were also seen for the structurally similar drug compound eplerenone.

Spironolactone has been used besides other Panx1-inhibitors to study the role of Panx1 in physiologic mechanisms. As such, spironolactone was utilized to examine the importance of Panx1 in cardiomyocytes in the context of non-ischemic heart failure (Pavelec et al. 2024) and to reveal a connection between Panx1 and the key transcription factor of the Wnt signalling pathway, β-catenin (Sayedyahossein et al. 2021). In vivo, spironolactone was shown to mitigate aortic inflammation by inhibition of endothelial cell-mediated ATP-release in mice (Ladd et al. 2023). In addition, the use of orthogonal Panx1-inhibitors including spironolactone, PBN and ^10^Panx1 helped to illuminate the involvement of Panx1 in the regulation of hypoxic pulmonary vasoconstriction (Grimmer et al. 2022). Intriguingly, not ATP release itself but modulation of the pulmonary artery smooth muscle cell (PASMC) Ca^2+^ response to hypoxia was the factor attributable to Panx1. In 2023, a novel cell extraction method for membrane proteins was developed and named Salipro (Drulyte et al. 2023). This method is supposed to facilitate extraction of transmembrane proteins in general and Panx1 in particular. Notably, the group determined K_D_ values for spironolactone and carbenoxolone and observed lower efficacies (spironolactone: 160 µM, CBX: > 256 µM) as compared to previously determined IC_50_ values (spironolactone: 7 µM, CBX: 5 µM). The explanation for this observation is the low specificity of both compounds resulting in more complex second-hand inhibition in addition to original Panx1 inhibition.

### Mefloquine

The anti-malaria drug mefloquine (Fig. 3) was reported to be a Panx1-inhibitor nearly twenty years ago (Iglesias et al. 2008). Both mefloquine and CBX inhibited Yo-Pro-1 uptake in J774 cells. As mentioned previously, CBX is to some extent able to differentiate between Panx1 and Cx43. Mefloquine, however, binds manyfold stronger to Panx1 than Cx43 (100% inhibition: 100 nM for Panx1 over 30 µM for Cx43) (Iglesias et al. 2008). After initial difficulties to reproduce mefloquine’s Panx1 inhibition, the same group discovered that different diastereomers of mefloquine exhibit distinct Panx1-inhibitory capacities (Iglesias et al. 2010). Recently, mefloquine has been used to uncover mechanistic insights into physiological pathways. As such, mefloquine was utilized to discover a new pathway for intravascular communication in skeletal muscle between capillaries and the upstream arterioles controlling their perfusion. This pathway is dependent on purinergic signalling with Panx1 transmitting vasodilatory signals from capillaries to arterioles (Lamb et al. 2021). Muscle contraction was shown to trigger various vasodilatatory pathways at the capillary level, which then mediate the transmission of signals to upstream arterioles (Lamb et al. 2024). In 2022, mefloquine as well as ^10^Panx1 helped to discover that dentinal sensitivity via intercellular odontoblast-neuron signal communication is dependent on Panx1, P2X3 receptor, Piezo1 and TRPA1 channel activation (Ohyama et al. 2022).

### Raptinal

**Fig. 4:**
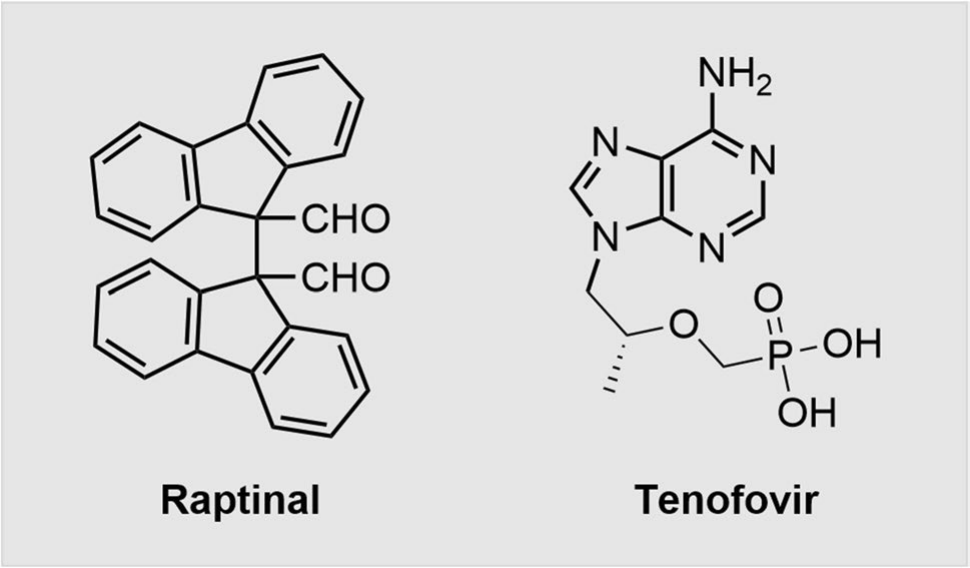
The two recently rediscovered Panx1-inhibitors raptinal and tenofovir possess interesting structural features. Tenofovir’s chemical structure contains both an acidic group (phosphoric acid) and an aromatic system (adenine), hence the Panx1-inhibitory effects are plausible. Raptinal on the other hand has an unusual structure with two highly reactive aldehyde groups

Raptinal (Fig. 4) rapidly induces caspase-dependent intrinsic apoptosis in multiple cell lines and in vivo systems (Palchaudhuri et al. 2015) and has recently been identified being a Panx1-inhibitor. Besides inducing apoptosis, raptinal was also shown to block the Panx1-dependent release of ATP as well as TO-PRO-3 uptake in apoptotic cells (Santavanond et al. 2024). Hence, raptinal represents the first known compound being able to simultaneously induce apoptosis and inhibit Panx1 channels. Other pro-apoptotic agents such as anti-Fas or UV light did not show comparable effects on dye uptake. Additionally, reduction of TO-PRO-3 uptake was also discovered in primary mouse thymocytes and in zebrafish embryonic cells upon raptinal treatment. Raptinal’s Panx1-inhibitory properties seem independent from its pro-apoptotic effects since blockade of apoptosis did not influence raptinal-induced Panx1 inhibition. The raptinal-mediated Panx1 inhibitory effects were further compared to those of trovafloxacin and CBX. In Panx1-inhibition assays, raptinal was more effective than trovafloxacin long-term but entirely ineffective short-term. Interestingly, while Panx1 inhibition by trovafloxacin and CBX was reversible and dye uptake could be restored after washout steps, raptinal inhibited Panx1 by an irreversible mechanism, indicating a unique binding mechanism (Santavanond et al. 2024). The authors discuss the possibility of covalent binding of raptinal to Panx1, and this idea is in fact plausible given raptinal’s unusual and highly reactive chemical structure with two aldehyde groups.

### Tenofovir

Tenofovir (Fig. 4), an antiviral agent that had been in clinical trials for the treatment of hepatitis B and HIV, inhibited ATP release from RAW264.7 cells in a dose-dependent fashion (IC_50_ = 2 µM) (Feig et al. 2017). This inhibition was abolished in Panx1-deficient cells, indicating that tenofovir is indeed a Panx1-inhibitor. The chemical structure with an acidic group and an aromatic system also resembles common structural features of inhibitors like PBN or NPPB (see below). To our knowledge the Panx1-inhibitory properties of tenofovir have surprisingly not been covered by a review before and should therefore be kept in mind for potential future applications in pannexin research.

### Other Panx-1-inhibitors

Besides the well-known Panx1-inhibitors that have been used repeatedly for biological studies, others have not gained much attention recently. NPPB (5-Nitro-2-(3-phenylpropylamino)benzoic acid), a chloride channel blocker, which blocks ATP release in ciliary epithelial cells (Mitchell et al. 1998) was also tested for its Panx1 inhibitory activity (Silverman et al. 2008). Interestingly, the compound was a remarkably strong inhibitor (IC_50_ = 50 µM) of Panx1 and is suspected to attack the same binding site as probenecid since the inhibitory effects of the two drugs were not amplified in combination. Contrary to PBN, NPPB (Fig. 3) also inhibited both Cx46 and Cx32E143, which might have hampered its use in pannexin research over the past 10 years. Instead, it served as a template in structure/activity relationship (SAR) studies (Crocetti et al. 2021). A set of chloride channel blockers was analysed with respect to Panx1 activity and, besides NPPB, disodium 4,4’-diisothiocyanatostilbene-2,2’-disulfonate (DIDS), 4-acetoamido-4’-isothiocyanato-stilbene-2,2’-disulfonate (SITS) and indanyloxyacetic acid 94 (IAA-94) (Fig. 3 and 6) showed remarkable efficacy (Ma et al. 2009). Interestingly, unlike the other two, DIDS also inhibited the P2X_7_ receptor. It is worth mentioning, that DIDS has later been shown to inhibit paracrine activation of P2Y2Rs and Panx1-channels in glia-like type II cells from the carotid body (Zhang et al. 2012). In addition, DIDS reduced ATP-release in polyethyleneimine-induced immune responses, which were analysed in human bronchial epithelial cells and in mice. The authors, however, attributed the observed effects to the inhibition of VDAC-1 (Srisomboon et al. 2021).

After the surprising discovery of the negative feedback loop of ATP (Qiu and Dahl 2009) and its even more active derivative BzATP, both inhibitors have served as helpful tools in the elucidation of Panx1 structure and physiology at that time. Interestingly, BzATP is a manyfold stronger Panx1-inhibitor than ATP, however, it displays lower selectivity and is an established P2X_7_R-inhibitor as well (Fig. 3).

**Fig. 5:**
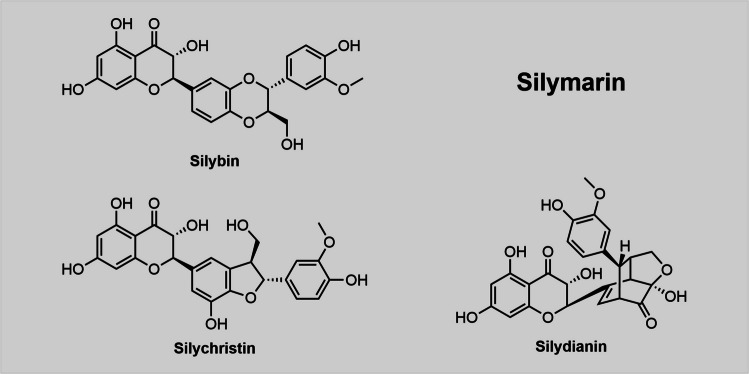
Silymarin is a mixture of various chemical components including silybin, silychristin and silydianin. These three compounds share structural similarities and are suspected to be Panx1-inhibitors

Three ingredient compounds of silymarin (Fig. 5), namely silybin, silychristin and silydianin, lowered extracellular ATP levels in three different cell lines (Yıldız et al. 2025). The road to elucidate their Panx1-inhibitory profile, however, was rocky as all three compounds interfered with fluorescence signals in certain assays.

**Fig. 6:**
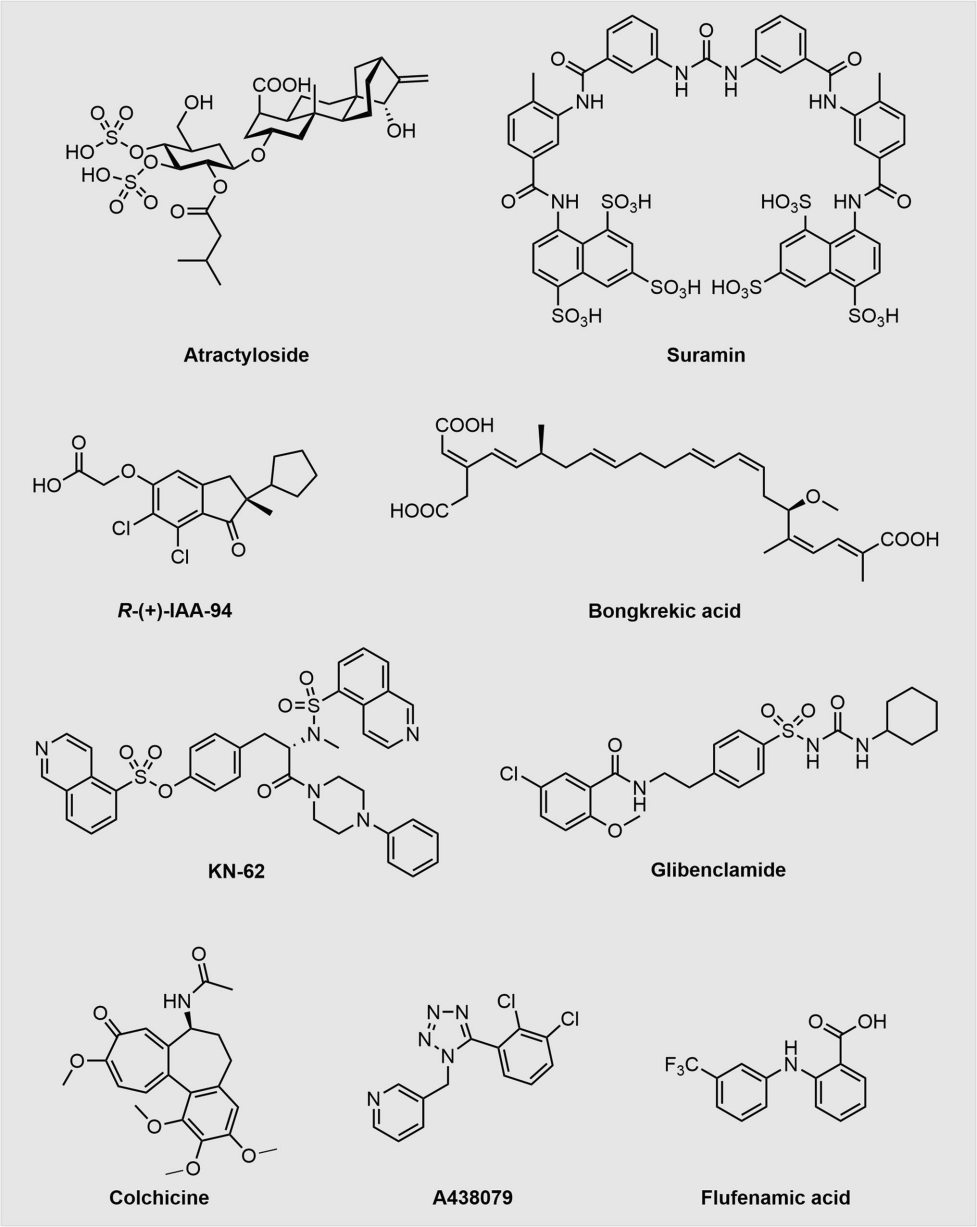
The chemical diversity of some lesser-known Panx1-inhibitors provides opportunities to pursue drug discovery. Their chemical structures indicate that the inhibition of Panx1 is not necessarily bound to certain structural motifs

Two compounds, which are not exclusively known for their Panx1 activity but rather as inhibitors of the mitochondrial ATP efflux are bongkrekic acid and atractyloside (Fig. 6). Nevertheless, their Panx1 activity was determined and thereafter compared to the much stronger inhibitory activity on the mitochondrial pore (Dahl et al. 2013). To distinguish between both effects, the dye uptake studies were carried out with erythrocytes, in which mitochondrial activity can obviously be neglected. In the same report, suramin, KN62, A438079, flufenamic acid, colchicine and glycyrrhetinic acid also showed Panx1 inhibitory effects (Fig. 6). Notably, the same group discovered the Panx1-inhibitory capacity of glibenclamide (Qiu et al. 2011). Another Panx1-inhibitor that, to our knowledge, surprisingly has not been covered by any Panx1-inhibitor review is lanthanum (Nielsen et al. 2020).

The use of La^3+^ did not significantly attenuate Panx1-mediated currents, however ethidium uptake was in fact reduced. Later, lanthanum as well as CBX and PBN were utilised to alleviate inward-currents in thapsigargin-stimulated hippocampal neurons (Patil et al. 2022). Thapsigargin is known to inhibit the sarcoendoplasmic reticulum calcium adenosine triphosphatase (SERCA) pump. Table 1 displays examples of previously applied concentrations of Panx1 inhibitors in both in vitro and in vivo studies. The list of Panx1-inhibitors mentioned herein is certainly not complete and a significant number of little-known inhibitors has already been covered by numerous other reviews (Dahl et al. 2013; Willebrords et al. 2017; Navis et al. 2020). In the following part, we will therefore be focussing on current developments of new inhibitors that were tailored solely for the purpose of Panx1-inhibition.

**Table 1:** Generic concentrations of Panx1 inhibitors in in vitro and in vivo applications. In vivo species and cell lines are given in brackets

Panx1 inhibitor	Applied Panx1 inhibitory concentrations	References
PBN	0.5—2 mM (HCT116)	(Fierro-Arenas et al. 2024)
	50 mg/kg (mouse)	(Kwok et al. 2024)
CBX	50 µM (NRK-52E)	(Yin et al. 2022)
	100 µM (rat)	(Zepeda-Morales et al. 2025)
BBG	1 µM (Xenopus oocytes)	(Qiu and Dahl 2009)
	50 mg/kg (rat)	(Mendes et al. 2023)
BB-FCF	10 µM (Xenopus oocytes)	(Wang et al. 2013)
	100 µM (rat)	(Beckel et al. 2015)
ATP	200 µM (Xenopus oocytes)	(Qiu and Dahl 2009)
BzATP	20 µM (Xenopus oocytes)	(Qiu and Dahl 2009)
Trovafloxacin	10 µM (CD8^+^ T cells)	(Vardam-Kaur et al. 2024)
	4.2 mg/kg (mouse)	(Vardam-Kaur et al. 2024)
Spironolactone	20 µM (HEK293T)	(Good et al. 2018)
	40 or 90 mg/kg (mouse)	(Good et al. 2018)
Mefloquine	10 nM (J774)	(Iglesias et al. 2008)
	0.03 mg/g (rat)	(Ohyama et al. 2022)
Raptinal	10 µM (Jurkat T cells)	(Santavanond et al. 2024)
Tenofovir	0.1–10 µM (RAW264.7)	(Feig et al. 2017)
	75 mg/kg (mouse)	(Feig et al. 2017)
NPPB	50–200 µM (Xenopus oocytes)	(Silverman et al. 2008)
DIDS	1–1000 µM (Xenopus oocytes)	(Ma et al. 2009)
SITS	1–1000 µM (Xenopus oocytes)	(Ma et al. 2009)
IAA-94	1–1000 µM (Xenopus oocytes)	(Ma et al. 2009)
Bongkrekic acid	4 µM (frog erythrocytes)	(Dahl et al. 2013)
Atractyloside	50 µM (frog erythrocytes)	(Dahl et al. 2013)
Glibenclamide	100 µM (Xenopus oocytes)	(Qiu et al. 2011)
^10^Panx1	200 µM (RAW264.7)	(Salgado et al. 2025)
	100 µM (mouse)	(Furlow et al. 2015)

## Medicinal Chemistry in Panx1 research

A frequent problem of Panx1-inhibitors has been their low selectivity and the abundance of numerous undesired co-targets. This issue has profound consequences on the outcome of scientific studies and makes the inhibitors unpredictable with respect to their application as drugs. Studies that rely on Panx1 inhibition by a non-selective inhibitor always face the challenge of discriminating between Panx1-mediated or side-target-mediated results. Additionally, observed side effects, such as hepatotoxicity of the Panx1-inhibitor trovafloxacin, cannot be assigned to Panx1 inhibition per se. A well-established practice in Panx1 research has been the use of complementary inhibitors with diverging side targets. As such, the combination of PBN and CBX has been a prominent example to rule out side target mediated observations. BB-FCF, albeit not designed specifically to target Panx1, is probably the most selective Panx1-inhibitor. Yet the fact that it is a strongly absorbing dye complicates numerous assays based on photometry. Therefore, the development of more selective as well as more effective Panx1-inhibitors is highly desirable. Efforts have been taken to overcome these problems and peptide as well as non-peptide compounds have been developed to achieve a more selective Panx1-inhibition to study physiological issues, such as Panx1 regulation, gating mechanisms, molecular structure, and therapeutic applications.

### Peptide inhibitors

#### ^***10***^***Panx1***

The first inhibitors developed specifically for Panx1 were peptide sequences of putatively critical areas within the Panx1 protein, a concept well known from connexin hemichannel research (Warner et al. 1995; Dermietzel et al. 2003). A prominent example is ^10^Panx1, which was developed in the early years of pannexin research (Pelegrin and Surprenant 2006). The group tailored the peptide to establish Panx1 as the large pore originally thought to be part of the P2X_7_ receptor. The peptide comprises the conserved amino acid sequence WRQAAFVDSY and was therefore suspected to mimic W74-Y83 of the first extracellular loop (EL1) of Panx1. Although its binding mode has not been elucidated yet, electrophysiological studies indicate that a sequence-dependent binding is unlikely as connexin-mimetic peptides achieved only slightly lower efficacies for Panx1-inhibition (Wang et al. 2007). Scrambled peptide sequences showed clearly lower efficacies, however, they did show some inhibition and Wang et al. therefore suggested a steric effect. This hypothesis is also in accordance with the observed stronger influence on dye uptake and ATP release (Pelegrin and Surprenant 2006) compared to electrophysiology studies as these molecules are bulkier than single-atom ions like chloride. An alternative hypothesis, however, may be interferences with the channel assembly (Lamouroux et al. 2023). Notably, Cx46 currents were also significantly attenuated by ^10^Panx1 in electrophysiological studies. Thus, in our view the pannexin-mimetic peptide cannot be considered a Panx1-selective inhibitor as suggested by others (García-Rojas et al. 2023; Salgado et al. 2025). In most studies, ^10^Panx1 has been used to inhibit Panx1-mediated ATP release or dye uptake. As such, the importance of C-tail truncation in pathological processes and Ca^2+^ influx was investigated (Salgado et al. 2024). The same group also observed an influence of ^10^Panx1 treatment on immune responses to Poly (I:C) incubation of murine RAW264.7 cells, which was evaluated by TNF-α and IL-1β levels (Salgado et al. 2025). In conclusion, ^10^Panx1 is a helpful tool in Panx1 research and may further contribute to Panx1 structure revelations because of its potentially different binding mode. However, it remains important to carefully evaluate the obtained results and exclude any possible side target effects, for example by use of a second or even third orthogonal Panx1-inhibitor.

The possibility of a different binding mechanism converts ^10^Panx1 into an attractive target for drug discovery. In a first lead optimisation approach starting from ^10^Panx1, it was possible to generate compounds with a higher plasma stability and efficacy (Caufriez et al. 2023). Important structural features within the decapeptide structure were initially identified by fragment analysis and replacement of all amino acids with their d-analogue, which intriguingly did not alter Panx1 activity but increased plasma stability half-life from 2 min for ^10^Panx1 up to more than 24 h. Inverting single amino acids, however, decreased the activity dramatically, possibly due to alterations of the peptide’s secondary structure. Upon stepwise replacement of amino acids, both Q76 and D81 were identified being most critical for Panx1-inhibition. Additionally, the replacement of F79 with the bulkier biphenylic amino acid 1-naphthyl-l-alanine (1’Nal) increased the potency and similar effects were observed for the replacement of D81 by the prolonged amino acid glutamate. Furthermore, changes regarding Y83 had a positive effect on efficacy as well. Three modifications, namely phenylalanine, tryptophan and 1’Nal replacements, increased Panx1-efficacy with the phenylalanine replacement being the most active derivative. In turn, the hydroxy group of Y84 might not be necessary for Panx1-inhibition.

**Fig. 7:**
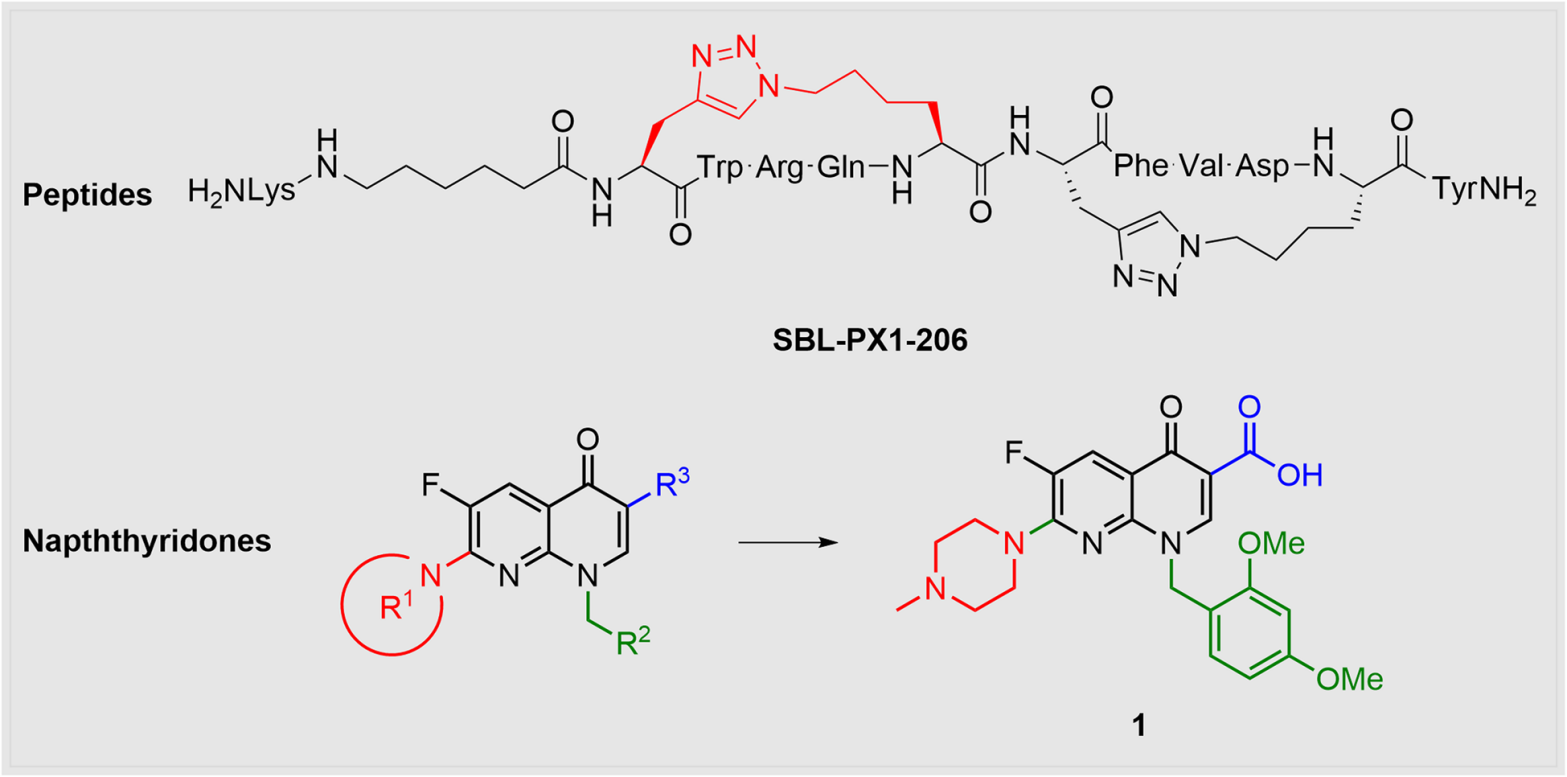
The most promising “double stapled” analogue of ^10^Panx1 (SBL-PX1-206) is depicted with a coloured triazol linker. The general naphthyridone structure displays the three variable residues, named R^1^ (red), R^2^ (green) and R^3^ (blue)

A second approach to improve ^10^Panx1 was pursued by the same group as they applied a prominent principle in drug development: They synthesised “stapled” ^10^Panx1 analogues to increase both stability and efficacy by immobilising the peptide compound (Lamouroux et al. 2023). This principle is based on entropic effects and is usually carried out by introducing double bonds or by cyclisations. Since the ^10^Panx1 sequence is part of an α-helical segment, the group developed a method to bring ^10^Panx1 into a fixed helical structure, which is the synthesis of single- and double-stapled macrocyclic triazol compounds through a copper-catalysed click-reaction. They systematically connected one amino acid with the respective *i* + *4* amino acid by a triazol linker; the four supposedly critical amino acids W74, R75, Q76 and D81, however, were not modified. Panx1-inhibitory capacity was determined by measurements of hypoosmotic stress (HOS)-induced ATP release and of Yo-Pro-1 uptake in B16-BL6 cells. Notably, Yo-Pro-1 uptake was significantly less influenced by these peptide inhibitors than ATP release. Plasma stability was not sufficient with the mono-stapled analogues as there remained sensitive cleavage sites outside the macrocycle. Therefore, a second macrocycle, leading to “double-stapled” peptides, was introduced. As this also led to decreased water solubility, DMSO was used to solubilise the new derivatives and, notably, they exerted elevated Panx1-inhibition. To overcome solubility issues, a 6-aminohexanoic acid residue was N-terminally introduced to provide the optimised peptide SBL-PX1-206 (Fig. 7). Eventually, overall plasma stability could be increased by double-stapling the peptide with 20% of the compound still being intact after 24 h. The newly synthesised peptides were further investigated regarding their anti-inflammatory properties and promising results were observed, especially for the double-stapled analogue. An issue that remains to be met is the selectivity of the new ^10^Panx1 derivatives.

#### *PxIL2P*

Αlpha1-adrenergic receptor (α1AR) stimulation has been under thorough investigation as a Panx1 channel activator. To determine the responsible regions for this regulatory effect a set of peptides from different Panx1 regions were utilised including PxIL2P, which is a short peptide sequence mimicking the intracellular loop (IL2) (Billaud et al. 2015). This peptide comprises the sequence between K191 and K200 as well as a TAT-sequence to help with plasma membrane permeation. PxIL2P was shown to inhibit α1AR-dependent vasoconstriction and ATP release from intact arteries. After progressive alanine substitution, the ^198^YLK sequence of Panx1 proved essential for channel activation by α1AR stimulation. The abundance of a tyrosine allows the speculation about a potential phosphorylation site being linked to channel activation. In a study about the role of Panx1 in renin-expressing cells, treatment of As4.1 cells with PxIL2P resulted in a reduction of intracellular calcium concentrations (DeLalio et al. 2020). Hence, the authors concluded that in this context Panx1 channels act by controlling intracellular calcium rather than ATP-release. This assumption was further supported by findings of Panx1 in endothelial cells being involved in controlling intracellular calcium concentrations (Yang et al. 2020). PxIL2P has also been used recently alongside other inhibitors both in vitro and in vivo to further study the consequences of Panx1 inhibition. As such, PxIL2P was demonstrated to inhibit vasoconstriction in smooth muscle cells. Additionally, a reduction in atrial arrhythmia burden in mice pre-treated with PxIL2P was observed (Dunaway et al. 2023; Mezache et al. 2023).

#### ***Panx1***_***308***_

Another segment of Panx1 that has been targeted by customised peptides is the C-terminal tail (Weilinger et al. 2012). The group developed a mimetic peptide connected to a TAT-sequence to reach the intracellular CT-region, which they later called TAT-Panx1_308_ (Weilinger et al. 2016). The conserved amino acid sequence comprises Y308, a suspected phosphorylation site, which may be involved in channel activation. The compound was reported to be efficacious in reducing stroke-induced cortical lesions and sensorimotor deficits in vivo (Weilinger et al. 2016). In a second report, the group also showed that TAT-Panx1_308_ induced increased spontaneous excitatory synaptic currents (sEPSC) frequency upon paired-pulse stimulation (PPS) (Bialecki et al. 2020). Panx1_308_ was further used in migraine studies in mice (Dehghani et al. 2023).

In conclusion, the development of mimetic peptides opens a new approach of targeting Panx1 by a yet unclear binding mechanism. While most Panx1-inhibitors have been suspected to target Panx1 in the proximity of the selectivity filter, both PxIL2P and Panx1_308_ introduce new potential binding sites for Panx1 inhibition in the intracellular domains. Further work needs to be done to verify the selectivity and the actual binding mode of these peptides, and it should be kept in mind that simply mimicking a peptide sequence does not guarantee selectivity. However, the tremendous efforts and the creativity to use stapling as a tool to modify ^10^Panx1 led to improved efficacies and plasma stabilities and eventually shows that mimetic peptides may be an alternative starting point for successful drug development.

### Trovafloxacin analogues

Trovafloxacin has not been very popular in pannexin research due to its significant hepatotoxic effects in vivo. However, its high substrate selectivity made it attractive for SAR studies and promising compounds were created in a detailed lead optimisation study of trovafloxacin derivatives for the treatment of inflammatory bowel disease (IBD) (Hsueh et al. 2025). The group decided to select trovafloxacin as starting point, because of its outstandingly high affinity to Panx1 without targeting Panx2 or Cx43. To determine inhibitory effects on Panx1 channels, they focused on orthogonal methods e. g. whole-cell voltage clamp recordings, fluorescent dye-uptake assays, ATP release assays and apoptotic body formation assays. Starting from trovafloxacin, three residues branching from the naphthyridone core structure were diversified. The authors also addressed possible hepatotoxic effects referring to Sun et al., who discovered in 2008 that trovafloxacin-induced liver toxicity is likely to originate from the cyclopropylamine structure (Sun et al. 2008). Hence, Hsueh et al. replaced the cyclopropylamine structure with the presumedly less toxic bioisosteres morpholine, methyl piperazine, pyrrolidine or with a simple hydroxy group.

The SAR studies revealed that sizable groups are essential for an effective binding at the R^1^ position (Fig. 7). Interestingly, the R^2^ position, which has mostly been examined by using different aromatic residues, was only slightly affected when replaced by a cyclohexyl derivative, thus preventing π-stacking interactions. Replacing fluorine atoms of R^2^ by methoxy groups and ergo increasing both the electron density in the system and the steric hindrance led to an increased inhibition of To-Pro-3 uptake. Additionally, insertion of a methylene or even an ethylene group between the nitrogen atom and the aromatic system (Fig. 7, green) further enhanced the inhibition and therefore implies that the influence of R^2^ is mainly based on steric reasons or hydrophobicity rather than electronic properties. Complete removal of R^2^ resulted in an almost entire loss of activity. Any alterations for group R^3^ significantly decreased the inhibition and underscored the assumption that the carboxylic acid contributes substantially to the binding mode.

Whole-cell voltage clamp recordings in HEK293T cells indicated that the optimised lead compound **1** (Fig. 7) had an IC_50_ of 0.73 µM at negative membrane potentials (–50 mV), a more than fivefold higher efficacy than trovafloxacin. Besides lead optimisation, the group also examined the potential binding site W74 by molecular modelling calculations. In a TUNEL assay, compound **1** seemed to attenuate colitis symptoms in mice in a more effective fashion than trovafloxacin.

With respect to the potential hepatotoxic effects of the newly synthesised compound, the group carried out a topoisomerase assay and observed that both trovafloxacin and the optimised derivative did not exert considerable effects on topoisomerase activity in vitro. These results indicate that the topoisomerase assay might not be the most meaningful method to determine hepatotoxicity since, as already mentioned, trovafloxacin is well known to be hepatotoxic. The authors state the absence of hepatotoxic tissue damage in treated mice, although this again is no proof for drug safety. In mouse models, trovafloxacin had only been shown to be hepatotoxic in combination with LPS (Poulsen et al. 2014), which means that the herein applied model was not likely to reveal liver tissue damages in the first place. Further studies will have to be carried out to examine possible side effects in vitro and in vivo.

### Indole derivatives

**Fig. 8:**
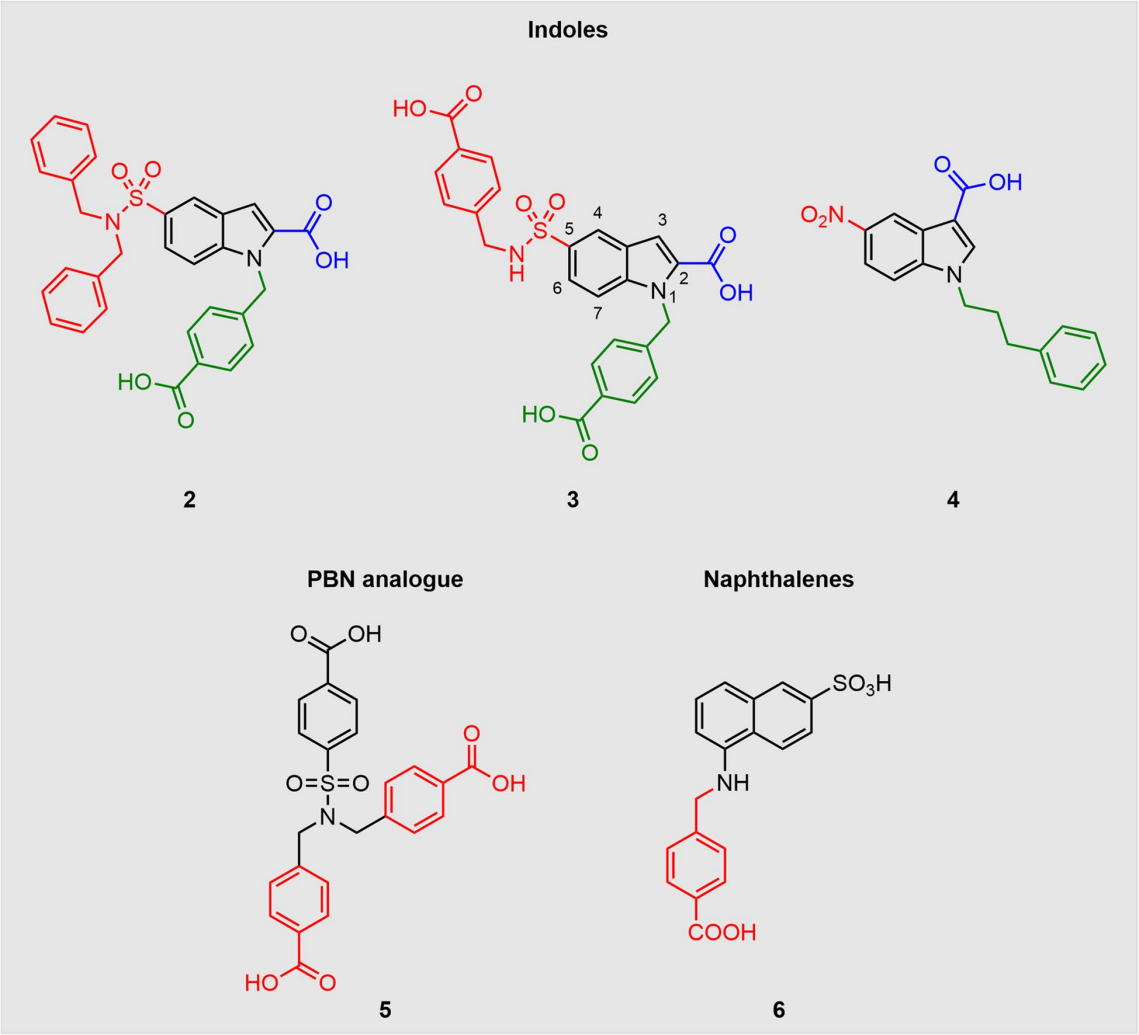
Highly active tailored Panx1-inhibitors derived from indoles, and naphthalenes are displayed. Compound **5** is a PBN analogue with increased potency to Panx1. For clarity reasons, the atom numbering is shown for compound **3**. The different colours indicate the functional groups varied in the respective studies

Indole-derived compounds were the first non-peptide Panx1-inhibitors with no prior applications in other contexts (Crocetti et al. 2021). Upon screening a library of relatively simple heterocyclic scaffolds, the group decided to choose the indole-type compounds for further studies, mainly due to limited synthetic complexity. They then inserted characteristic groups, which are also present in NPPB and PBN, such as a sulfonamide, carboxylic acid, or nitro group. All new products were screened for their Panx1 activity by voltage clamp experiments with *Xenopus laevis* oocytes. To differentiate between Panx1 and P2X_7_R inhibition, candidate compounds were also tested for P2X_7_R-mediated membrane currents induced by ATP. Insertion of a sulfonamide group at position 5 (Fig. 8) together with the introduction of an additional acid group significantly increased Panx1 blocking efficacy by reaching 100% inhibition. The abundance of secondary and tertiary sulfonamides appeared more effective than primary sulfonamides. Moreover, the introduction of two carboxylic acid groups on the secondary sulfonamide indicated a rise in activity. Intriguingly, the replacement of the sulfonamide group with an amine resulted in dramatic loss of activity. The two most effective compounds **2** and **3** (Fig. 8) exhibited an IC_50_ of 0.25 µM and 0.8 µM respectively. Additionally, the group tested some of the compounds with respect to selectivity by applying them to oocytes expressing either P2X_7_R or Cx32E_1_43. While the dibenzylated compound **2** showed some activity to P2X_7_R, the carboxybenzylated derivative **3** abolished any P2X_7_R activity. Interestingly, the less potent compound **4** exerted a great selectivity for Panx1 over P2X_7_R. As pannexin channels play a crucial role in pain, the two selective Panx1-inhibitors **3** and **4** were investigated in vivo against pain hypersensitivity induced by oxaliplatin. In mice, an active dose of 0.3 nmol of compound **3**, injected intrathecally, induced an enhancement of pain threshold starting after 45 min. Hypersensitivity was even entirely reverted at an active dose of 1 nmol. Nitro compound **4** showed a similar profile with slightly lower efficacy.

### Naphthalene, pyrazole and PBN derivatives

Besides indoles, naphthalenes and pyrazoles were chosen for further SAR studies and groups with promising results in the indole study were incorporated (Crocetti et al. 2022). In addition, a special place in the study was reserved for the new PBN analogue **5** with two carboxylic acid groups. Voltage clamp experiments revealed that chain prolongation of the monobenzylated naphthalene derivative had a positive effect on inhibition. By far the biggest impact, however, was made by the introduction of a carboxylic acid group in *para*-position, raising the inhibitory effect from 31.7% to 97% for compound **6**. Remarkably, the new PBN analogue **5** with additional carboxybenzyl groups displayed 93.7% of inhibition, while PBN only reached 29% inhibition. While the naphthalene derivatives achieved promising results, the pyrazoles did not reach inhibition rates beyond 43%. Thus, the PBN derivative **5** (IC_50_ = 1.2 µM) reached a remarkable value compared to other established Panx1 blockers (PBN = 150 µM, CBX = 10 µM). The two most promising compounds **5** and **6** (Fig. 8) were tested in a mouse model of neuropathic pain as described in the indole-themed study, and both demonstrated an enhancement of pain threshold in a dose-dependent manner.

### Quinolone and quinoline derivatives

**Fig. 9:**
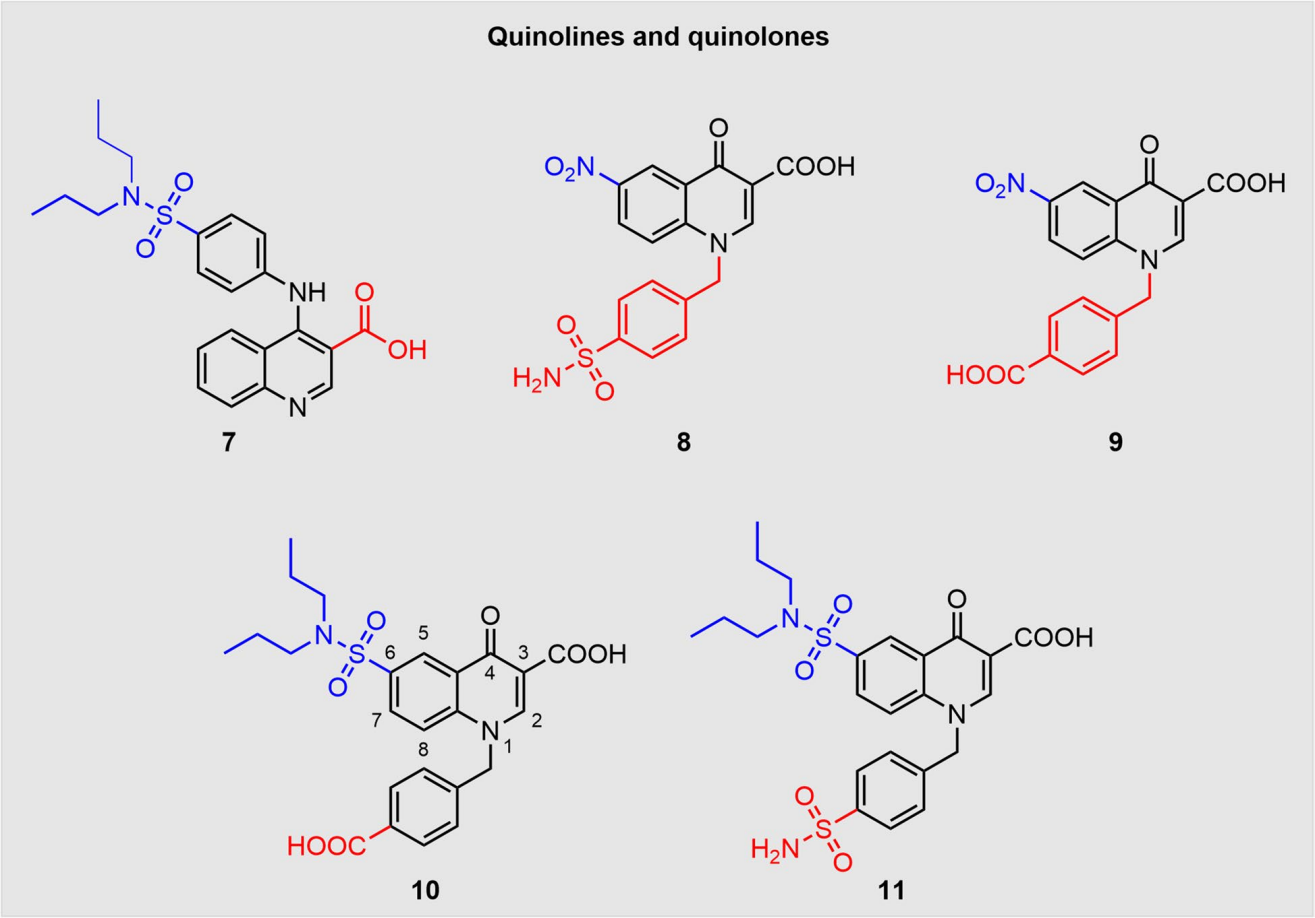
Quinoline- (compound **7**) and quinolone-derived (compounds **8**, **9**, **10** and **11**) inhibitors of Panx1 are displayed. For clarity reasons, the atom numbering is shown for compound **10**. The different colours indicate the functional groups varied in the respective studies

While previous drug developments had structural elements derived from trovafloxacin, PBN, NPPB or BB-FCF, another study put the focus on mefloquine derivatives (Crocetti et al. 2023). New quinoline- and quinolone-type Panx1-inhibitors were therefore developed (Fig. 9). The introduction of a primary sulfonamide or a carboxylic acid in *para*-position had a profoundly positive effect on the inhibition reaching 80% and 89%, respectively. In case of the quinolone derivatives, carboxylic acids and tertiary sulfonamides had positively affected inhibition. The four most promising compounds including structures **7**, **8** and **9** (Fig. 9) were subjected to dose–response studies as well as selectivity studies for P2X_7_R and connexins. IC_50_ values were in the range of 1.5 µM-15.5 µM and all four compounds did not interact with connexins while compounds **8** and** 9** abolished P2X_7_R inhibition. Contrary to the two preceding SAR studies, the group investigated the plasma stability of the corresponding ester derivatives of the most active compounds. Surprisingly, the ester derivatives were observed to be remarkably stable, which makes them less appropriate for anticipated prodrug approaches.

The quinolone portfolio was expanded by the introduction of a sulfonamide (Crocetti et al. 2025), which further increased their activity. In addition, new mefloquine analogues with a broad range of substitutions in the 4-position were synthesised (Fig. 9). Biological results were obtained through patch-clamp analysis of CHO cells transfected with mPanx1 cDNA plasmids. While the new mefloquine-derived analogues did not exhibit satisfactory Panx1-inhibition, the modified quinolones achieved promising results with the best result even reaching 100% inhibition. Tertiary sulfonamides in combination with a carboxylic acid group resulted in a strong Panx1-inhibition comparable to the potent compound **9**. Intriguingly, while in the aforementioned SAR studies the carboxylic acid group reached better results than the respective primary sulfonamide, in case of compounds **10** and **11**, the combination of a dipropyl sulfonamide with a primary sulfonamide was able to improve activity up to 100% as compared to 85% for the respective carboxylic acid derivative (Fig. 9). First experiments in a model of renal cyst development with mpkCCD_cl4_ cells displayed that treatment with 3 µM of compound **11** during the growth period (3 weeks) significantly attenuated cyst development.

The development of new Panx1-inhibitors by conventional Medicinal Chemistry is still a young field of research. To our knowledge, only two research groups have engaged with the development of non-peptide Panx1-inhibitors and tremendous efforts have led to remarkable results so far. The development of new Panx1-inhibitors is not only one step closer to a promising drug but is also a versatile tool to further investigate the structure and physiology of Panx1, especially when combining it with molecular modelling.

### Antibodies and nanobodies

Besides the synthesis of the traditional chemical inhibitors, antibodies have found their way into Panx1-inhibitor research, following a concept originally developed with the aim of connexin hemichannel inhibition (Dermietzel et al. 2003). The first antibody to be developed was the Panx1-blocking antibody HRB454 or α-Panx1-EL, which recognises the WRQAAFVDSY sequence in the first extracellular loop of Panx1 (Molica et al. 2019). Additionally, the group also generated the control antibody HRB460 or α-Panx1-CT, directed against part of the intracellular Panx1-CT. HRB454 reduced collagen-induced ATP release from human platelets by half compared to the control experiment. In a mouse model, HRB 454 was able to delay the tail bleeding time and venous thromboembolism.

A fascinating method to improve Panx1-specific antibodies was the development of nanobodies (Van Campenhout et al. 2023). The three newly generated nanobodies recognise both murine and human Panx1 in a nanomolar range and exerted significant Panx1-blocking capacities in ATP release assays using hPanx1-overexpressing DUBCA cells. To further assess anti-inflammatory effects, IL-1β levels were determined in an LPS/ATP-mediated inflammation model using RAW264.7 cells. Intriguingly, only two of the nanobodies, namely Nb3 and Nb9, showed anti-inflammatory effects in vitro. Their application in in vivo biodistribution studies showed increased nanobody uptake in salivary glands and stomach. Additionally, anti-inflammatory effects were examined in vivo by ^99m^Tc radiolabelling experiments. The observed reduced serum levels of cytokines, likely to be associated with the NLRP3 inflammasome, make nanobodies seem promising as anti-inflammatory agents in vivo.

In 2025, both binding potential and cytotoxic effects of seven different Panx1-specific nanobodies have been investigated (Rusiecka et al. 2025). In those experiments, six of these recognised Panx1 in endothelial cells with the nanobody Nb9 displaying the strongest binding (K_D_ = 1.9 nM). Lactate dehydrogenase (LDH) levels were measured to assess cytotoxicity of the nanobodies in vitro and, intriguingly, no cell death was observed after 30 min of nanobody treatment. The ability of the newly created nanobodies to affect ATP release through Panx1 was investigated in EA.hy926 cells and their specificity was confirmed by control experiments using Panx1-deficient H9c2 cells. In the context of cardiac ischemia/reperfusion (I/R) treatment, Nb1 and Nb9 were the most promising candidates. In studies on the inhibition of neutrophil adhesion to endothelial cells they showed a more than twofold inhibition compared to probenecid. Interestingly, Nb9 was demonstrated to have a potentially cardiotoxic effect both ex vivo and in vivo, while, in contrast, Nb1 did not exhibit any negative effects on cardiac function.

## Conclusion

Since the early days of pannexin research, inhibitors have been indispensable tools to enable Panx1 research. The ongoing struggle with the selectivity of Panx1-inhibitors and the resulting need for more selective compounds has finally been met by interdisciplinary researchers. Panx1 is a fundamental component in a wide range of pathological conditions and an important target for medical treatment. Off-target effects could be diminished with more selective compounds and drug development is the method of choice to develop tailored molecules. The correct validation of Panx1 specificity, however, remains indispensable for newly developed drug compounds as well as repurposed drugs. The combination of non-orthogonal or unselective Panx1 inhibitors may lead to inconclusive results, while e. g. the use of Panx1-deficient animals displays a more reliable alternative verification method. To further facilitate drug development, in vivo pharmacokinetic and pharmacodynamic studies will be necessary, especially for the determination of potentially labile functional groups within the Panx1 inhibitor as well as for metabolism or even toxicity issues, that had already been decisive factors for drugs like trovafloxacin. The promising results received from drug discovery so far will hopefully be groundbreaking for the development not only of additional Panx1-inhibitors but also of compounds that specifically target Panx2 and Panx3. Molecular dynamics simulations may further contribute to elucidate binding mechanisms of Panx1 and ergo of the gating mechanisms leading to both reversible and irreversible ATP release.

## Data Availability

No, I do not have any research data outside the submitted manuscript file.
